# Neglected Respiratory Toxicity Caused by Cancer Therapy

**DOI:** 10.2174/1874306400701010001

**Published:** 2007-07-30

**Authors:** Christian Domingo, Jorge Roig

**Affiliations:** 1 Servei de Pneumologia. Hospital de Sabadell (Corporació Parc Taulí)- Departament of Medicine, Universitat Autònoma de Barcelona (UAB) Sabadell (Barcelona/Spain). Anatomy and Physiology Department, Facultat de Ciències de la Salut, Universitat Internacional de Catalunya (UIC). Barcelona, Spain; 2 Unitat de Pneumologia. Hospital Nostra Senyora de Meritxell. Escaldes-Engordany, Principality of Andorra

**Keywords:** Non-chemotherapy-induced lung toxicity, administration route toxicity, lung injury, cancer treatment toxicity.

## Abstract

When a patient with lung cancer presents non-specific respiratory symptoms there are many diagnostic options. Chemotherapy is the cornerstone of treatment in many stages of lung cancer and its toxicity is well known. The main priority is to prevent life-threatening diseases such as lung infection, which can be treated successfully if a prompt, accurate diagnosis is given. Drug-induced pulmonary disease must be avoided at all costs but it is also important to avoid side-effects of drugs which do not directly interfere with respiratory physiology but may impair gas exchange. This review highlights the risks and characteristics of non-cytostatic-induced lung toxicity caused by agents that have been commonly used to treat cancer in recent decades. Physicians should be alert to the possibility of this neglected non-chemotherapy-induced lung toxicity in cancer patients, since early withdrawal of the offending drug is mandatory.

## 1 INTRODUCTION

Since the first report of a link between busulfan and pulmonary toxicity was published in 1961 [1], the literature on lung disease induced by cytotoxic drugs has grown steadily [2]. As the number of drugs used in cancer chemotherapy increases, the incidence of pulmonary toxic effects may rise even further, especially since patients with cancer now survive longer and their lung cell populations are exposed to cytotoxic agents for longer periods of time.

Oncologists often have to consider a broad differential diagnosis when lung cancer patients under cytostatic treatment present non-specific respiratory symptoms and radiographic shadowing [3-5]. Because of their prognostic and therapeutic implications the following etiologies should be assessed first in the initial diagnostic approach: lung infection, caused either by conventional pathogens or by atypical microorganisms, malignancy-related thromboembolic pulmonary disease, local tumor progression, iatrogenic intraalveolar hemorrhage, radiotherapy-induced adverse effects, transfusion reactions, postoperative complications in patients who have undergone thoracic surgery, oxygen toxicity, and finally drug-induced pulmonary toxicity. In this last case, most patients are receiving a wide array of drugs other than cytostatics which may cause lung toxicity [6]. Once the clinician has raised the suspicion of drug toxicity, the classic criteria proposed by Karch and Lasagna [7] should be applied so as to ensure accurate diagnosis (Table 1).

The aim of this article is to review the other frequently neglected side-effects of cancer treatment in which the drugs involved do not interfere with parenchymal lung physiology but may eventually have repercussions on gas exchange.

## 2 METHODOLOGY

First we undertook a large-scale review of pulmonary toxicity caused by cytotoxic drugs [2] and selected the chemotherapeutic drugs responsible for respiratory disturbances not related to their cytotoxic effect. We then reviewed the different non-cytotoxic drugs currently used to treat malignancies, and selected those with potential lung toxicity. Finally, we examined the side-effects related to the administration route used. We searched the PubMed database for all prospective and retrospective studies reporting these items and referenced those providing relevant information.

The paper is divided into three sections. In the first we discuss the mechanisms of how chemotherapy indirectly causing parenchymal lung toxicity may eventually alter respiratory function (Table 2); in the second we refer to the pulmonary toxicity caused by non-cytotoxic drugs used in the treatment of malignancies (Table 3) associated with the administration of the chemotherapy protocol. In the third, we consider the side-effects due to the drug administration route (Table 4).

## 3 SIDE-EFFECTS OF CHEMOTHERAPY INDIRECTLY CAUSING PARENCHYMAL LUNG TOXICITY

### 3.1 Respiratory Muscle Impairment

Procarbazine, cytarabine, chlorambucil and above all vincristine may cause neuropathies which can affect respiratory muscle function [8]. The primary manifestations are usually sensory, but drug-induced motor or sensorimotor neuropathies also occur, most commonly caused by vincristine. Most of these case are mild or even subclinical symmetric distal neuropathies. It is not clear whether the respiratory muscles are ever involved, though some of the many cases of idiopathic phrenic nerve palsies are likely to be drug-induced [9]. The pathogenic mechanism seems to be neurotubular damage. This adverse event may be particularly serious when anesthesia is administered to these patients [10].

### 3.2 Acute Encephalopathy and Respiratory Depression (Ifosfamide and Methotrexate)

This side-effect merits special attention, given the high prevalence of chronic obstructive lung disease and sleep apnea-hypopnea syndrome. Both entities can cause chronic hypercapnia which may deteriorate with the addition of an extra offending agent that favors respiratory depression [8].

Direct pulmonary toxicity due to ifosfamide is very rare but possible [11]. The risk of CNS depression following ifosfamide therapy (about 12%) [12-13] should be considered in patients with advanced chronic obstructive lung disease, a common association in lung cancer, since somnolence may worsen hypercapnia and enhance encephalopathy. Caution is also needed in the case of underlying central or obstructive sleep apnea syndrome, since its prevalence in the general population is relatively high.

Methotrexate (MTX) is a cell cycle-specific agent that inhibits the enzyme dihydrofolate reductase, preventing the conversion of folic acid to tetrahydrofolic acid and inhibiting cell replication. MTX crosses the blood-brain barrier and can be administered intravenously and *via* an intrathecal route to eradicate leukemic cells from the CNS and prevent CNS recurrence. This is why chemotherapeutic regimens include methotrexate for acute lymphocytic leukemia treatment. Both high-dose intravenous MTX and intrathecal MTX are associated with demyelination, white matter necrosis, loss of oligodendroglia, axonal swelling, microcystic encephalomalacia, and atrophy relatively selective for the deep cerebral white matter. A diminished choline-to-creatine ratio has been reported in children with MTX-related neurotoxicity; this may be related to disturbances of myelin metabolism or inhibition of glucose metabolism induced by MTX [14].

Acute MTX neurotoxicity usually results in stroke-like symptoms, such as aphasia, weakness, sensory deficits, ataxia, and seizures. Several cases of MTX-induced acute encephalopathy have been reported [15-19], presenting with confusion and even coma with high-dose methotrexate therapy. As with ifosfamide, clinicians should bear in mind the possibility of respiratory compromise in severe COPD.

### 3.3 Non-Cardiogenic Pulmonary Edema

Non-cardiogenic pulmonary edema may be caused by several drugs and multiple mechanisms In some cases, CNS toxicity due to intrathecal methotrexate may be the cause [20-21]. Less frequently, this methotrexate administration route has been associated with interstitial pneumonitis [22]. Non-cardiogenic pulmonary edema may also be the result of increased permeability. This has been reported with IL-2 infusions as well as with docetaxel [23]. Sirolimus (previously called rapamycin) is a macrolide antibiotic isolated from the fungus *Streptomyces hygroscopicus.* It has potent anticandida activity, but subsequent studies have revealed impressive antitumor and immunosuppressive activities as well [24]. The drug is used to prevent rejection of organs and bone marrow transplants and is also under investigation as a treatment for cancer. Among its side-effects, pulmonary edema or distress syndrome have occasionally been described [25].

### 3.4 Pleural Effusion

Docetaxel may cause pleural effusion by increasing permeability. However, although large effusions may cause severe respiratory compromise, some authors do not consider this to be a classic pulmonary toxicity. Occasionally, methotrexate has also been found to cause pleurisy [26].

## 4 PULMONARY TOXICITY DUE TO NON-CYTOTOXIC DRUGS USED TO TREAT MALIGNANCIES

Many non-cytotoxic drugs can cause pulmonary toxicity. In this section we mention three groups of drugs, the first two of which are widely prescribed in cancer patients. We use the common toxicity criteria grading [27].

### 4.1 Granulocyte Colony-Stimulating Factor (G-CSF)

These drugs are frequently prescribed to hasten recovery from cancer chemotherapy-induced neutropenia. Some have been reported to induce pulmonary edema. Pegfilgrastim is a long-acting G-CSF, recently approved by the Food and Drug Administration [28], and lenograstim is the glycosylated recombinant form of human granulocyte colony stimulating factor [29].

### 4.2 Monoclonal Antibodies

Monoclonal antibodies are a new class of non-cytotoxic agents targeted at specific receptors on cancer cells. In addition to their direct cellular effects, antibodies can carry substances such as radioactive isotopes, toxins, and antineoplastic agents to the targeted cells. Five monoclonal antibodies-rituximab, trastuzumab, gemtuzumab ozogamicin, alemtuzumab, and ibritumomab tiuxetan -- are available for clinical use. Monoclonal antibodies currently under study include edrecolomab and tositumomab [30-31].

Trastuzumab is a humanized anti-HER2 mAB which achieves response rates of 15-40% when used as a single agent in patients with metastatic HER2-overexpressing breast cancer, and prolongs survival when added to chemotherapy in first line therapy in these patients [32]. The major side-effects include pulmonary [33] and cardiac toxicity. Fleming *et al.* [32]. treated 45 patients with trastuzumab and IL2, a regimen that is no longer given today; grade 3 pulmonary toxicity or higher was found in five patients and attributed to intermediate-dose IL2 in three. Of the seven grade 2 pulmonary toxicities, six occurred in 21 patients known to have lung metastasis Several trastuzumab first-dose infusion-related reactions with pulmonary components were also found. Byrd *et al.* [34] observed that the five patients they treated with trastuzumab developed severe pulmonary infusion-related toxicity. Occasionally, organizing pneumonia has also been attributed to trastuzumab [35].

Rituximab is also known to cause pulmonary dysfunction, though probably less frequently than trastuzumab. Alveolar hemorrhage [36], acute pneumonitis and pulmonary fibrosis have been observed [37 -40]. Very occasionally, a desquamative alveolitis is also found [41].

Recently, Biehn *et al.* [42] reported the first case of bronchiolitis obliterans organizing pneumonia caused by rituximab. However, **l**ung toxicity seems to be quite rare. In a recent study, Kanelli *et al.* [43] observed only one case of toxicity in a cohort of 27 patients.

Gemtuzumab ozogamicin was approved in 2000 by the FDA. Its major side-effect is hepatotoxicity (31% of patients show abnormal liver enzymes) but adult respiratory distress syndrome (ARDS) has also been reported [44]. Alentuzumab causes immunosuppression, increasing the risk of infections [31] and possibly ARDS [45]. Novel monoclonal antibodies such as tositumomab, edrecolomab and cetuximab appear promising but are still under evaluation [46-49].

### 4.3 All-Trans Retinoic Acid

Retinoids are natural or synthetic derivatives of vitamin A. All-trans retinoic acid has clinical applications in acute promyelocytic leukemia. More than half the patients treated with this drug experience retinoic acid syndrome, a disorder characterized by fluid retention, weight gain, respiratory distress, radiographic pulmonary infiltrates, hectic fever and pleural or pericardial effusion [50].

### 4.4 New Iatrogenic Risk Factors for Deep Venous Thrombosis and/or Pulmonary Embolism

It has long been recognized that patients with malignancies have an increased risk of thromboembolism [51], sometimes preceding cancer diagnosis or, more commonly, as complications. In addition to the well-known risk factors such as chemotherapy, hormonal therapy and tumor type and stage, other therapeutic strategies have been added to the list of risk factors for developing deep venous thrombosis and/or pulmonary embolism.

#### 4.4.1 Erythropoietin

Erythropoietin is the primary hematopoietic growth factor for erythropoiesis. It has proved effective in increasing hemoglobin levels in the majority of patients with cancer and anemia [52-53]. Erythropoietin may contribute synergically to thrombosis in this setting by increasing red cell mass and thus whole blood viscosity. It also results in reticulocytosis, which raises the proportion of young metabolically active red blood cells, thus increasing platelet reactivity *in vitro*. The drug has also been found to be synergistic with the platelet growth factor thrombopoietin for platelet activation and is associated with increased platelet reactivity and endothelial activation. Recent findings suggest that platelet-red cell interactions play a role in venous thrombosis. Taken together, these data suggest that erythropoietin may be thrombogenic. In a retrospective case-control study undertaken in 147 patients with carcinoma of the uterine, cervix or vagina treated with chemoradiotherapy, Wun *et al.* [54] found symptomatic thromboembolic disorders in 23%. Patients treated with recombinant human erythropoietin were 10 times more likely to develop a thrombosis than non-treated patients. Dusembery *et al.* also reported a rate of 20% (four out of twenty patients) [55].

#### 4.4.2 Megestrol Acetate

This drug is a progestational agent which is currently used as an appetite stimulant in patients suffering from cancer anorexia/cachexia syndrome [56-57]. In a retrospective review, Bolen *et al.* [58] found that more than 30% of their 18 nursing home residents treated with megestrol developed deep venous thrombosis. The controversy in the literature is evident since the results of Oberhoff *et al.* [59] did not provide any evidence of a thrombotic effect of high-dosage megestrol acetate amongst patients with advanced gynecological malignancies. These authors concluded that the increase in the incidence of thrombosis observed in other reports may be the consequence of other risk factors such as tumour-induced hypercoagulability, simultaneous chemotherapy or other individual thrombosis risk factors. The treatment appears to have been used safely in other studies [56-57].

#### 4.4.3 Thalidomide

Thalidomide is a glutamic acid derivative drug with antiangiogenic and tumor necrosis factor (TNF) blocking agent properties that result in anti-inflammatory and immunodepressive effects. The drug is considered an adjuvant immunosuppressant [60]. Since increased bone marrow vascularity has a poor prognosis in myeloma, the efficacy of thalidomide has been evaluated in patients with refractory disease by several research groups [61 -62]. Common side-effects of thalidomide are mild, dose-related and reversible and include constipation, fatigue, paresthesias and dry skin. More rarely, the drug has been found to be associated with an increased risk of thromboembolism when used in patients with malignancy [63]. Although single-agent thalidomide has minimal prothrombogenic activity, its combination with cytotoxic chemotherapy is associated with a significant increase in the risk of deep venous thrombosis [64]. Current recommendations are that clinicians consider using long-term aspirin [65] or even coumarin concomitantly with thalidomide in this situation. According to Baz *et al.* 65] daily low-dose aspirin (81 mg) administered orally reduced the incidence of vascular thrombosis without increasing bleeding complications. Moreover, Scarpace *et al.* [66] also reported that two out of four patients who developed arterial thromboses treated with thalidomide were receiving concomitant anticoagulation with aspirin and warfarin. Finally, since erythropoietin increases the risk of vascular thrombosis, it could be argued that the concomitant use of this drug and thalidomide could have an additive effect on hypercoagulability, but according to Galli *et al.* [67] erythropoietin does not seem to increase the risk of thrombosis in myeloma patients receiving thalidomide.

### 4.5 Epidermal Growth Factor Receptor Tyrosine Kinase Inhibitors (Gefitinib and Erlotinib)

Recent advances in cancer biology have led to the identification of several potential molecular targets that play a key role in cancer development and progression. Selective targeting of cancer cells on the basis of their molecular phenotype can provide effective anticancer activity, avoiding the side effects commonly induced by cytotoxic chemotherapy. Among these targets, the epidermal growth factor receptor (EGFR) has been widely investigated in different human malignancies due to its critical role in cancer proliferation and survival. The epidermal growth factor receptor (EGFR) is involved in cancer progression and development and, being overexpressed in a variety of human malignancies, is an attractive target for selective anticancer therapy. EGFR tyrosine kinase inhibitors (TKIs) produce dramatic and durable responses in a fraction of non-small cell lung cancer patients [68–69].

Lung toxicity related to this tyrosine kinase activity inhibitor is very uncommon and usually mild [70]. Worldwide, the incidence of interstitial lung disease is <1% [2]. However, severe pulmonary reactions have also been reported [71].

## 5 SIDE-EFFECTS DUE TO DRUG ADMINISTRATION ROUTE

### 5.1 Bronchial Artery Infusion of Cytostatic Drugs

Though not a new therapy, the effectiveness of the infusion of cytotoxic drugs into the bronchial artery has been reconsidered during the past decade for treatment of central advanced and early staged lung cancer [72] as well as for lung metastasis, although the instances reported in the latter case are fewer [73]. Though the technique allows the infusion of high-density chemotherapeutics into objective limited lesions [72] side-effects are not eliminated. In their study, Osaki *et al.* [72] treated seven patients, three of whom subsequently presented severe bronchial ulcers and one of whom died of pulmonary hemorrhage. Suzuki *et al.* [73] recently reported a massive hemoptysis from a bronchial pulmonary arterial fistula in a patient with lung metastasis. Lung hemorrhages usually occur between one and three months after bronchial artery infusion. Other side-effects include esophageal ulceration, spinal cord damage, and bronchial esophageal fistulas.

### 5.2 Intrathoracic Extravasation of Cytostatics

Most extravasation accidents involve the limbs However few data are available regarding intrathoracic extravasation of cytostatic agents administered in central venous lines. To date, only seven cases have been reported [74], in one child and six adults. In three cases the perforations were acute and were entirely procedure-related. Pain and fever were the prominent symptoms and leukocytosis was present in three patients. Computed tomography chest scan was the diagnostic method used. Late perforations may be related to an increased concentration of cytostatics due to elevated venous pressure which may favor the erosion of the vessel wall. Although there is no commonly accepted treatment at present, the standard administration of dalteparin/warfarin can allow the central line to remain in situ with little risk of line failure or recurrence/extension of the DVT [75]. Recent guidelines do not recommend antithrombotic prophylaxis (AP) to prevent catheter-related thrombosis in cancer patients with a central line. In their study, Fagnani *et al.* observed no difference in catheter-related thrombosis in patients given AP or not (2.8% *vs* 2.2% respectively). Systemic venous thromboembolism (including deep and superficial thromboses and pulmonary embolism) was less frequent with AP (4% versus 8.2%), mortality was also lower (25% versus 44%, but only advanced cancer, not AP, was found to be significantly associated with mortality. No major bleeding was recorded with AP. So current AP schedules do not appear to prevent catheter-related thrombosis. Systemic venous thromboembolism and patients’ mortality, however, appeared lower after prophylaxis [76].

### 5.3 Venous Thromboembolism Associated to Central Venous Catheters

The first long-term central venous catheter (CVC) was introduced in 1973 by Boviac for parenteral nutrition. In the early 1980s, totally implantable port systems were introduced in clinical practice. Among early pulmonary complications are pneumothorax, hemothorax and air embolism (estimated rate ranging between 0.3% and 12%) [77]. CVC-related deep venous thrombosis are among late complications. The reported incidence of symptomatic catheter-related deep venous thrombosis in adults varies from 0.3% to 28% while the incidence of venographically demonstrated thrombosis ranges between 27 and 66%. The incidence with subcutaneous ports seems to be lower, around 1% (2 out of 175 patients [78–79].

## CONCLUSIONS

Respiratory toxicity other than the classical chemotherapy-induced lung toxicity may have a substantial impact on the prognosis of cancer patients. Frequently, withdrawal of the drug will ensure recovery. Though in general the incidence of this type of toxicity is low, oncologists should be aware of its existence; if it remains unrecognized, the likelihood of a fatal outcome is substantially increased.

## Figures and Tables

**Table 1:** Assessment of Link Between Agent and Event*

	Definite	Probable	Possible	Conditional
*Appropriate temporal sequence*	Yes	Yes	Yes	Yes
*Known reaction to agent*	Yes	Yes	Yes	No
*Improved with dechallenge*^1^	Yes	Yes	Yes-No	No
*Relapse on rechallenge*^2^	Yes	?	?	?
*Event reasonably explained by clicinal state or other therapies*	No	No	Yes	No

* Modified from Karch and Lasagna [7].

1 The effects of stopping the drug or reducing the dose.

2 Relapse of the effects after restarting the drug.

**Table 2:** Mechanisms of Respiratory Impairment Caused by Chemotherapy Indirectly Causing Parenchymal Lung Toxicity

Respiratory muscle impairment Acute encephalopathy Non-cardiogenic pulmonary edema. Pleural effusion

**Table 3:** Non-Cytotoxic Drugs Used for the Treatment of Malignancies

Granulocyte colony-stimulating factor (G-CSF) Monoclonal antibodies All-trans retinoic acid Drugs favouring deep venous thrombosis/pulmonary embolism: Erythropoietin Megestrol acetate Thalidomide Epidermal growth factor receptor tyrosine kinase inhibitors: Gefitinib Erlotinib

**Table 4:** Side-Effects Due to Drug Administration Route

Bronchial artery infusion of cytostatic drugs Intrathoracic extravasation of cytostatics Venous thromboembolism associated to central venous catheters
